# Alginate–gelatin hydrogel supplemented with platelet concentrates can be used as bioinks for scaffold printing

**DOI:** 10.2478/abm-2023-0063

**Published:** 2023-10-26

**Authors:** Tuyet Thi Vi Le, Nghia Thi Hieu Phan, Ha Le Bao Tran

**Affiliations:** 1 Department of Physiology and Animal Biotechnology, Biology and Biotechnology Faculty, University of Science, Ho Chi Minh City 700000, Vietnam; 2 Laboratory of Tissue Engineering and Biomedical Materials, University of Science, Ho Chi Minh City 700000, Vietnam; 3 Vietnam National University, Ho Chi Minh City 700000, Vietnam

**Keywords:** bioinks, biosignals, growth factors, hydrogels, printing, scaffolds, tissue regeneration

## Abstract

**Background:**

Owing to the growing global demand for organ replacement and tissue regeneration, three-dimensional (3D) printing is widely recognized as an essential technology in tissue engineering. Biomaterials become a potential source of raw materials for printing ink by containing factors that promote tissue regeneration. Platelet concentrates are autologous biological products that are capable of doing that.

**Objectives:**

This study was carried out to create bioinks capable of providing biological signals by combining gelatin–alginate with platelet concentrates.

**Methods:**

This study combined platelet concentrates, including platelet-rich plasma (PRP) and platelet-rich fibrin (PRF), with gelatin and alginate to create bioinks. Bioink properties, including gelatinization and pH, were assessed before printing. After that, the scaffolds were done, and the growth factor (GF) release and cytotoxicity from these scaffolds were performed.

**Results:**

Results showed that all the three bioinks, including alginate–gelatin (AG), alginate–gelatin-PRP (AGP), and alginate–gelatin-PRF (AGF) were gelatinized right at the end of bioink fabrication and had a pH around 7. The scaffolds from bioinks supplemented with platelet concentrates secreted GFs that remained for 12 d, and the extracts from them were not cytotoxic for the L929 cell line.

**Conclusion:**

In summary, bioinks were made by combining AG with platelet concentrates and had properties suitable for creating scaffolds with cell-oriented grafts in the development of artificial tissues and organs.

Three-dimensional (3D) bioprinting technology is an additive manufacturing system using a 3D bioprinter and biomaterials to create 3D tissues for scaffold-based fabrications, including extrusion. This technique has obtained a lot of interest in regenerative medicine and tissue engineering as a cutting-edge way to replace, repair, or reconstruct damaged tissues or organs [1]. The bioinks used in 3D bioprinting are crucial components of the printing process. Many bioinks used for 3D tissue bioprinting include synthetic-based hydrogels, protein-based hydrogels, polysaccharide-based hydrogels, and decellularized extracellular matrix (dECM)-based hydrogels [2]. These hydrogels have highly biocompatible, modifiable mechanical and biodegradable properties and give a high resolution during printing [3]. They can be used alone or in various combinations to improve cell proliferation, metabolic activity, and tissue functions. However, rapid tissue degeneration and loss of biological activity were observed in artificial tissue structures derived from this technology [4, 5]. Researchers focus on developing sophisticated bioinks in combination form to overcome these limitations to improve their biological properties.

Alginate is widely applied in tissue engineering because of its availability, low cost, biocompatibility, and one-step gelation process [6]. In some research, gelatin is usually combined with alginate to take advantage of its biocompatible cell adhesion based on richness in arginine, glycine, and aspartic acid (RGD) [7]. However, bioinks from alginate–gelatin (AG) cannot provide any biological signals, so adding growth factors (GFs) during or after creating the scaffolds is necessary. It has been identified that after combining bioactive elements and bioinks, cell proliferation, extracellular matrix formation, and collagen production increased compared with hydrogels that did not include bioactive factors [8]. One of the most appealing sources of bioactive cues is platelet concentrates, including platelet-rich plasma (PRP) and platelet-rich fibrin (PRF), which contain a wide range of plasma proteins and GFs that promote cell proliferation, differentiation, improve extracellular matrix synthesis, and stimulate tissue regeneration. Platelet concentrates have been successfully used as a therapeutic agent in many fields. Obtaining GF preparations from platelet concentrate technology has great potential in regenerative medicine [9, 10]. Previous studies have shown that the physical properties may be tuned by loading the platelet concentrates into biomaterials, and the degradation rate was slow [10].

In recent years, several research groups have developed bioinks that combine the first generation of platelet concentrates with biomaterials. PRP has been examined more extensively in the creation of bioinks than PRF because of its advantage of being in a liquid state. PRP has been explored for various tissue engineering applications, such as cartilage tissue engineering [11, 12]. Besides, PRF has also been studied as bioink of the injectable type by obtaining liquid phase plasma after centrifugation, which is used in soft tissue regeneration [13]. In these studies, PRP and PRF were applied in the form of low biological activity, such as inactive or platelet poverty, respectively [5, 13]. This may have an impact on their effectiveness.

In the current study, both activated PRP and PRF in a liquid state are used to make bioinks that are combined with AG. This combination can improve the release characteristic of the products. For printing orientation of tissue replacement therapies, the pH of the bioinks, the gel condition, and the GF release capacity of the scaffolds after printing were investigated. Then, cytotoxicity experiments employing L929 cells were used to assess cytocompatibility in vitro of bioinks.

## Materials and methods

### Animals

In the Laboratory of Tissue Engineering and Biomedical Materials (University of Science, VNU-HCM), *Mus musculus* var. *Albino* mice (6–8 weeks old) were housed under temperature and light control (12-h light/dark cycle, 23–28 °C). Water and pellets were freely available to the mice. A total of 38 mice were used to collect approximately 50 mL of blood for all experiments. This study was approved by the Animal Care and Use Committee-University of Science (Certificate of Approval No. 580B/KHTN-ACUCUS), University of Science, VNU-HCM, Vietnam.

### Preparation of platelet concentrates

To prepare PRP, mouse cardiac blood (about 1.5 mL) was collected and centrifuged at 3,500 rpm for 3 min in a tube containing 150 μL of acid citrate dextrose (ACD) (BD Vacutainer). Approximately 600 μL of plasma was extracted immediately after centrifugation, and this volume was centrifuged for 22 min at 3,000 rpm. After centrifugation, approximately 400 μL of the supernatant was discarded. Then, the pellets were pipetted with the remaining solution (200 μL) of the plasma to obtain non-active PRP. The collected PRP was activated by 20 μL of 230 mM CaCl_2_ (Sigma-Aldrich) [14]. The PRP supernatant was then collected from the PRP gel pressing.

To create PRF, mouse cardiac blood was centrifuged at 3,500 rpm for 15 min immediately after collection without anticoagulants. The PRF product only exists in a strongly activated gel form (fibrin clot). Erythrocytes were removed, and the fibrin clot was collected in a separate tube. The PRF supernatant obtained from pressing PRF was then collected.

The PRP and PRF supernatants were stored at −20 °C until further use.

### Preparation of bioinks

Alginate (Sigma-Aldrich), gelatin (Sigma-Aldrich), and CaCl_2_ (Sigma-Aldrich) powders were dissolved in deionized water (Sigma-Aldrich), respectively. The AG bioink was prepared by mixing solutions of 10% (w/v) alginate, 10% (w/v) gelatin, and 70 mM CaCl_2_ in a ratio of 1:1:0.5 by volume, respectively. The bioinks containing platelet concentrates were AG-PRP (AGP) bioink and AG-PRF (AGF) bioink, which was created by adding PRP and PRF supernatants into the AG bioink (1:10, v/v), respectively. These bioinks were transferred to a 3 mL syringe, and the bubbles in the bioink were removed by centrifuging at 3,000 rpm for 5 min.

### Physicochemical properties

The gelation of both AG, AGP, and AGF bioinks was determined by inverting the tubes containing the bioinks. These bioinks were added to the vials and sealed with screw caps. These vials were incubated at room temperature, and at regular intervals, the vials were inverted to check for a sol–gel transition. To check the pH of the bioinks, litmus papers were used. This quick method was carried out in order to determine whether bioinks are suitable for cell survival.

### 3D printed construct fabrication

The scaffolds were created using a 3D bioprinter with an extrusion-based design. The Solidworks software was used to model a cube (20 mm × 20 mm × 1 mm). The bioprinter (Bio X, Cellink) was loaded with a 3-mL syringe containing AG, AGP, and AGF bioinks. The following settings were chosen for 3D bioprinting: a needle with a 250-μm inner diameter, platform room temperature, fill density of 10%, and four layers with a layer thickness of 250 μm. After printing, the AG, AGP, and AGF scaffolds were cross-linked by soaking in 0.1 M calcium chloride (Sigma-Aldrich) for 10 min and then washed three times in 1X PBS (Gibco).

### Cytotoxicity assays

The cytotoxicity of AG, AGP, and AGF scaffolds was assessed using an extract test followed by an MTT (3-[4-C-dimethylthiazol-2-yl]-2,5-diphenyl tetrazolium bromide, Sigma-Aldrich) assay, according to ISO 10993-5 [15]. Both basic medium (DMEM/F12 [Sigma-Aldrich], 1% penicillin/streptomycin [Sigma-Aldrich]), and cell culture medium (DMEM/F12 [Sigma-Aldrich], 10% FBS [Sigma-Aldrich], 1% penicillin/streptomycin [Sigma-Aldrich]) were used to obtain extracts. These two types of extracts were referred to as serum-containing extracts and serum-free extracts, respectively. Scaffolds had a surface area of about 3 cm^2^ and were incubated in 1 mL of these media at 37 °C for 24 h. After incubation, the scaffold extracts were collected. The L929 cell line was cultured in 96-well plates, where each well contained 10^4^ cells until 80% confluency. Then, 100 μL of extracts were applied to each of the 96-well plates, which were then incubated for 24 h at 37 °C in a humidified 5% CO_2_. The media (the cell culture medium or the basic medium) and 20% DMSO were used as negative and positive controls, respectively. After discarding the medium, each well was filled with 100 μL of 0.5 mg/mL MTT solution (Sigma-Aldrich) and incubated for 4 h to form formazan crystals. After carefully discarding the liquids in each well, 100 μL DMSO: Ethanol (Sigma-Aldrich) (1:1, v/v) was pipetted into each well, giving the cell culture plate a uniform color. A microplate reader (Biochrom EZ Read) was used to measure the absorbance at a wavelength of 570 nm. The same experiment was performed with a serum-free medium for extract collection. The relative growth rate (RGR) was calculated as

$$\text{The}\;\text{relative}\;\text{growth}\;\text{rate}\;(\text{RGR})\;\%=\frac{\text{Absorbance}\;\text{of}\;\text{treated}\;\text{wells}}{\text{Absorbance}\;\text{of}\;\text{cell}\;\text{control}}\times 100$$

### GF release assays

The release of GFs from AG, AGP, and AGF scaffolds was assessed over 12 d. Scaffold extracts were collected at selected time points, including days 1, 4, 8, and 12, and stored at −20 °C until further use. The scaffolds were incubated in DMEM-F12 medium at 37 °C for 24 h to produce scaffold extracts. After 24 h, the solution was collected (day 1). Next, fresh DMEM-F12 medium was added, and incubation was maintained until day 4 when the supernatant was collected (day 4). The same procedure covered the following two-time intervals, including days 8 and 12. PRP and PRF supernatants were used as the controls. The total GF release was measured using ELISA kits specific for platelet-derived growth factor-AB (PDGF-AB, RAB1857-1KT, Sigma-Aldrich), and vascular endothelial growth factor (VEGF, RAB0509-1KT, Sigma-Aldrich).

### Statistical analysis

IBM SPSS Statistics V22.0 software (IBM Corporation) was used in this study. The data were presented as Mean ± SD and two-tailed Student's t-tests, one sample t-test, and ANOVA test were performed to assess statistically significant between groups (*P* < 0.05).

## Results

### Physicochemical properties of bioinks

**Figure 1.**
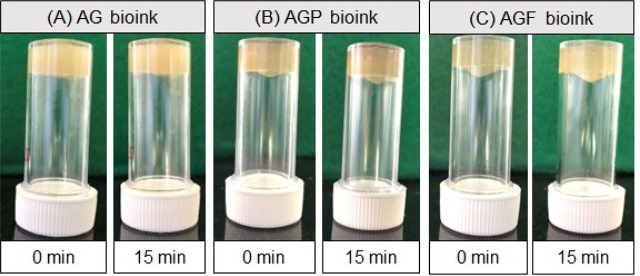
The sol–gel transition of bioinks over time through inversion test. **(A)** AG bioink; **(B)** AGP bioink; **(C)** AGF bioink. AG, alginate–gelatin; AGF, alginate–gelatin-PRF; AGP, alginate–gelatin-PRP.

**Figure 2.**
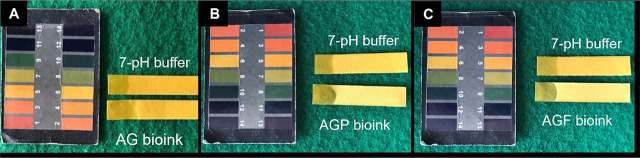
The pH of bioinks. **(A)** AG bioink; **(B)** AGP bioink; **(C)** AGF bioink. AG, alginate–gelatin; AGF, alginate–gelatin-PRF; AGP, alginate–gelatin-PRP.

The gelation of a bioink determines its sol–gel transition. The time it takes for a solution to turn into a gel is known as the gelation time. It is typically optimized to adjust for the rheological properties of bioinks. **Figure 1** shows the results of a survey conducted by the tube inversion method. We found that all of the bioinks, including AG, AGP, and AGF maintained their shape after the tubes were inverted. Gelation was presented immediately after blending bioink components, and this state was maintained for up to 15 min (enough time to complete print operations after that). The sol–gel transition times of three bioinks were not statistically different. On the contrary, to ensure the compatibility of composite materials from these bioinks, the bioinks’ pH needs to be controlled. By comparing with the color of the standard pH scale on litmus paper, the pH values of the AG bioink, AGP bioink, and AGF bioink all had a neutral pH of around 7 (**Figure 2**).

### In vitro GF release analyses

**Figure 3.**
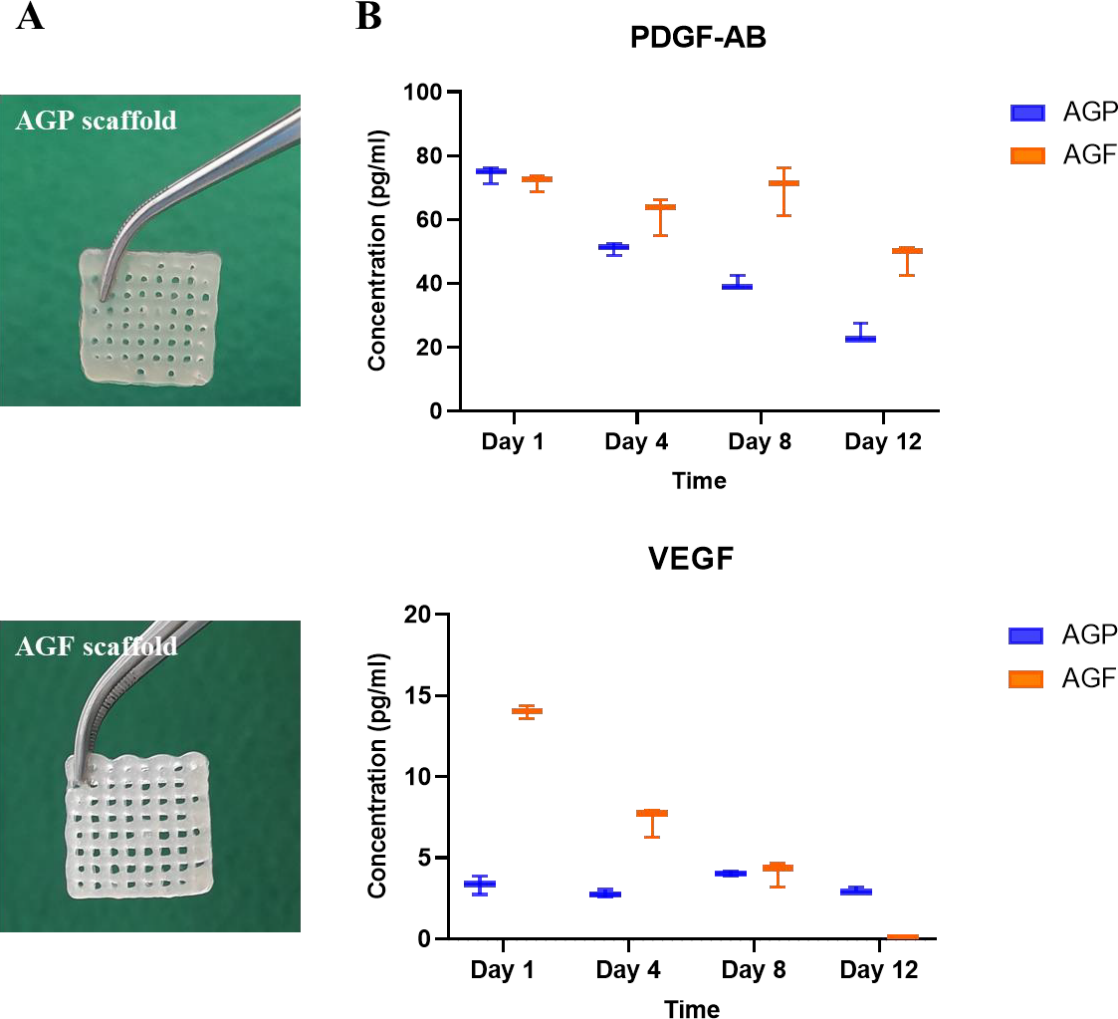
GFs release from scaffolds over time. **(A)** AGP and AGF scaffolds, **(B)** Growth factors release. AGF, alginate–gelatin-PRF; AGP, alginate–gelatin-PRP; GFs, growth factors; PDGF-AB; platelet-derived GF-AB; VEGF, vascular endothelial GF.

GF release is a required property of the scaffolds that plays an essential role in providing biologically active substances that promote tissue regeneration. This study demonstrated that the scaffolds that were compounded with a supernatant of 10% PRP or PRF could secrete PDGF-AB and VEGF for 12 d. However, there was a difference in the GF release between the two types of scaffolds (**Figure 3**).

On day 1, the PDGF-AB concentrations were the highest in all scaffolds for PDGF-AB release. At AGF scaffolds, this concentration was 71.67 ± 2.60 pg/mL, which remained constant for 8 d (*P* > 0.05) and decreased on day 12 (47.92 ± 4.73 pg/mL) (*P* < 0.05). However, not for AGP scaffolds, which decreased over time throughout the survey. On the contrary, PDGF-AB concentrations from PRP and PRF, which were added to AG hydrogel (10% in final hydrogels), were different. In PDGF-AB, concentration in the added-PRP was 89.37 ± 0.79 pg/mL, which was higher than in the added-PRF (61.22 ± 0.79 pg/mL) (*P* < 0.05). It was observed that the concentration of PDGF-AB secreted at AGF scaffolds was equivalent to that at 10% PRF added. It was different from AGP scaffolds when this GF was secreted at a lower concentration of 10% of the original PRP.

In contrast to PDGF-AB release, VEGF is stably secreted at AGP scaffolds, and the secretion of this GF tends to decrease rapidly over time at AGF scaffolds (**Figure 3**). Specifically, the VEGF concentrations from AGP scaffolds on days 1, 4, 8, and 12 were 3.33 ± 0.57 pg/mL, 2.79 ± 0.24 pg/mL, 4.03 ± 0.16 pg/mL, and 3.01 ± 0.19 pg/mL, respectively, and there were no statistically significant differences between them (*P* > 0.05). However, these concentrations were lower than the concentration of VEGF from PRP, which was added to AG hydrogel at a 10% concentration. Unlike the AGP scaffolds, the concentration of VEGF secreted by the AGF scaffolds on day 1 was 13.98 ± 0.40 pg/mL, which was higher than by 10% of the PRF added to the original hydrogels (8.62 ± 0.88 pg/mL) (*P* < 0.05). The concentration of this GF secreted from AGF scaffolds on day 8 of the investigation was equivalent to that from AGP scaffolds (*P*-value > 0.05). However, by day 12, VEGF secretion was not observed (**Figure 3**).

### Cytotoxicity analyses

**Figure 4.**
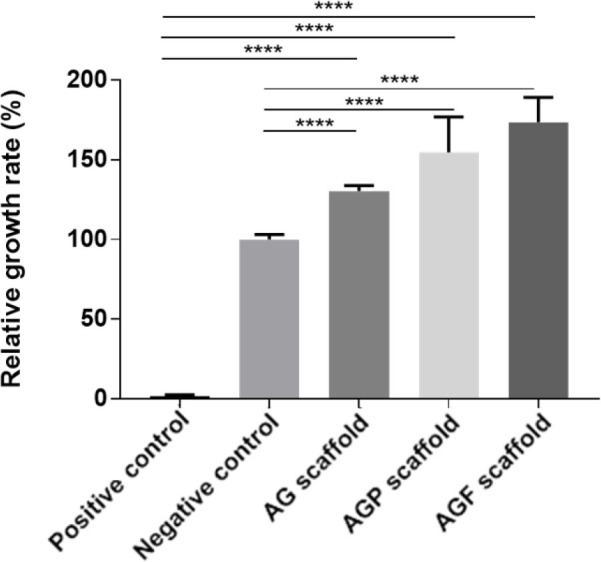
The RGR of cells in the serum-free extracts. ^****^P < 0.0001. AG, alginate–gelatin; AGF, alginate–gelatin-PRF; AGP, alginate–gelatin-PRP; RGR, relative growth rate.

The effect of AG, AGP, and AGF scaffolds on cell viability was evaluated through an MTT assay. Results showed that, in the serum-free extracts, the RGR of cells was highest in the AGF group, at 173.69 ± 15.48 (*P* < 0.05). This rate was found in the AG and AGP groups to be 130.36 ± 3.58 and 154.69 ± 22.21, respectively. Moreover, the cell growth in these three groups was higher than in the negative control group (*P* < 0.05). With the impact of 20% DMSO, the positive control had the lowest rate of cell growth, reaching 1.81 ± 0.78 (*P* < 0.05) (**Figure 4**).

**Figure 5.**
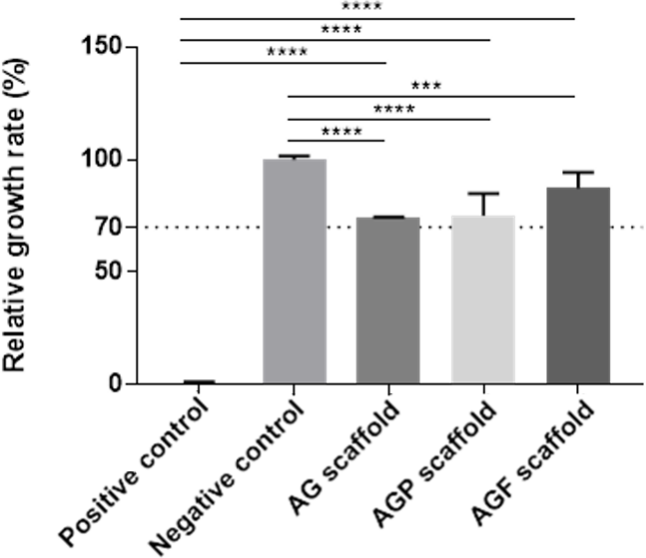
The RGR of cells in the serum-containing extracts. ^***^P < 0.001, ^****^P < 0.0001. AG, alginate–gelatin; AGF, alginate–gelatin-PRF; AGP, alginate–gelatin-PRP; RGR, relative growth rate.

On the contrary, in the serum-free extracts, the cell growth rates in the AG, AGP, and AGF groups were lower than in the negative control. These rates were 74.35 ± 0.16, 75.17 ± 9.83, and 86.75 ± 6.63, respectively. However, the cell growth rates in the PRP and PRF supplementation groups were higher than the positive control (*P* < 0.05). It was still over 70% (**Figure 5**). According to ISO 10993-5, if the RGR for the concentrations of the extracts is ≥70% of the positive control group, then the material shall be considered to be non-cytotoxic.

## Discussions

The development of tissue engineering utilizing 3D bioprinting technology has become popular in regenerative medicine, as it provides a novel therapeutic chance for numerous patients, such as developing tissue substitutes. Several bioink criteria are required for efficient tissue fabrication, such as shear thinning, mechanical properties, biodegradability, and biocompatibility. This study used bioink from gelatin, alginate, and mouse growth factors. Several characteristics of bioink were examined, including pH, bioink state, and GF secretion. The pH-neutral results demonstrated that these bioinks may be used to make scaffolds and would be safe for cell viability. Besides, the stable gelation status of bioink makes it easier to print and mold. The regular cross-linking in this bioink was due to alginate with CaCl_2_. The sodium ions in sodium alginate were substituted with calcium ions in this experiment. The ions can cross-link the polymer chains because each calcium ion can attach to two carboxylate groups, resulting in an insoluble, gel-like material [16]. In this research, CaCl_2_ was employed in two steps. The first step involved mixing 70 mM CaCl_2_ with hydrogel to create the ink, which was then loaded into a 3-mL syringe and used for printing structures. The reason for adding 70 mM CaCl_2_ is to increase the gelatinousness of the inks based on our exploratory studies. In the second step, the printed scaffolds were crosslinked with 100 mM CaCl_2_ to increase their stability. The concentration of CaCl_2_ used here is predicted to be safe for cells, because a study has reported that when the calcium content in the bath is greater than 100 mM, excellent cell viability can be achieved when using the alginate-based bioink [17]. Moreover, other research papers have also utilized the 100 mM CaCl_2_ solution to apply post-crosslinking to scaffolds [18, 19].

In vitro and in vivo, various GFs have been demonstrated to stimulate cell migratory, proliferative, and differentiation activity in additive and synergistic ways, supporting greater healing activity [20]. ELISA results showed that the AGP and AGF scaffolds could secrete the GFs PDGF and VEGF during the 12 d of the survey. PDGF and VEGF are two of the many GFs that control cell development and division. It is essential for blood vessel formation (angiogenesis), which produces new blood vessels from existing blood vessel tissue [21, 22]. Consequently, including PDGF-AB and VEGF in the AGP and AGF scaffolds might promote cell proliferation.

Moreover, the results of evaluating the ability to secrete GFs showed that there were differences in the secreting capacity of AGP and AGF, in which the concentration of PDGF and VEGF that was secreted from AGF was higher than from AGP, but depending on the type of GF, the secretory effects of two different types of scaffolds are required. The PDGF-AB and VEGF GFs released from the AGF scaffold on days 1, 4, and 8 were higher than the GF level in the 10% PRF solution. This may be related to the stability of the GF in a diffusible state. GFs typically exhibit low stability, a brief circulating half-life, and a fast rate of cellular internalization when present in a diffusible state [23, 24]. Therefore, the amount of released GF into the environment may have undergone degradation and denaturation. Incorporating the GFs into hydrogel scaffolds can extend the in vivo half-life of the GF [25]. On day 12, the GF released into the environment decreased. This can be explained, as previously discussed, by the degradation of the GFs, and the other reason is the low remaining level of GFs within the structure. It can be seen that the AG bioink structure can capture the GFs from PRP and PRF supernatants, which helps these factors maintain the release ability over time. The concentration of GFs released into the environment is influenced by the degradation of scaffolds. Faster degradation leads to a higher concentration of GFs in the environment. GA scaffolds take several weeks to degrade, depending on the type and concentration of the cross-linking agent used. The use of a cross-linking agent also improves the mechanical stability of the scaffolds compared to those without one [26, 27].

It is known that cell viability can be affected by any of the ingredients of the ink formulation and printing parameters [28]. In the current study, we demonstrated the viability of L929 cells when exposed to the scaffold extracts with and without PRP and PRF supernatants. In the serum-free extracts, AG scaffolds were identified to promote cell growth. Furthermore, when PRP and PRF supernatants were added to AG ink, cells proliferated effectively in scaffold extract when no serum was present. In the presence of serum-containing extracts, this was reversible. Many studies indicated that GFs require sustained glucose metabolism to promote cell survival. Cells growing in low GF concentrations are less susceptible to GF withdrawal-induced cell death [29]. In AGP and AGF extracts, the volume of GFs in serum and those released from scaffolds increase the concentration of GFs in the culture medium. However, the duration of GFs in serum is short and rapidly degraded [24, 30], resulting in a rapid decrease in GF concentration in the medium. Although GFs are essential components for cell development [24], cells developing in greater GF concentrations are more vulnerable to death if the GF is suddenly decreased [31].

## Conclusion

In this study, the sol–gel transition of bioinks, including AG, AGP, and AGF was determined. After mixing, the ink transitioned into a gel-like state, which is suitable for the printing process, and this state was maintained for at least 15 min. Besides, these bioinks have a pH of around 7, which is suitable for cell survival. On the contrary, scaffolds that were compounded with the PRP or PRF supernatant secreted GFs such as PDGF-AB and VEGF until day 12. Moreover, neither PRP nor PRF was cytotoxic when used for scaffolding in the serum-free extracts. However, the RGR decreased in the serum-containing extracts, but these extracts were still nontoxic to cells in the groups. Further studies on the use of this formulation in the formation of multicellular tissue constructions are ongoing.
